# Nickel-Catalyzed Radical Mechanisms: Informing Cross-Coupling for Synthesizing Non-Canonical Biomolecules

**DOI:** 10.1021/acs.accounts.3c00588

**Published:** 2023-11-30

**Authors:** Gregory A. Dawson, Ethan H. Spielvogel, Tianning Diao

**Affiliations:** Department of Chemistry, New York University, 100 Washington Square East, New York, New York 10003, United States

## Abstract

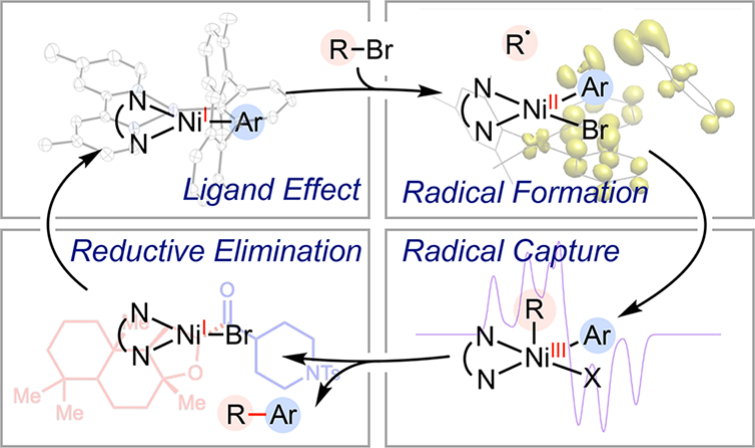

Nickel excels at facilitating selective radical chemistry, playing a pivotal role in metalloenzyme catalysis and modern cross-coupling reactions. Radicals, being nonpolar and neutral, exhibit orthogonal reactivity to nucleophilic and basic functional groups commonly present in biomolecules. Harnessing this compatibility, we delve into the application of nickel-catalyzed radical pathways in the synthesis of noncanonical peptides and carbohydrates, critical for chemical biology studies and drug discovery.

We previously characterized a sequential reduction mechanism that accounts for chemoselectivity in cross-electrophile coupling reactions. This catalytic cycle begins with nickel(I)-mediated radical generation from alkyl halides, followed by carbon radical capture by nickel(II) complexes, and concludes with reductive elimination. These steps resonate with mechanistic proposals in nickel-catalyzed cross-coupling, photoredox, and electrocatalytic reactions. Herein, we present our insights into each step involving radicals, including initiation, propagation, termination, and the nuances of kinetics, origins of selectivity, and ligand effects.

Radical generation from C(sp^3^) electrophiles via one-electron oxidative addition with low-valent nickel radical intermediates provides the basis for stereoconvergent and cross-electrophile couplings. Our electroanalytical studies elucidate a concerted halogen atom abstraction mechanism, where electron transfer is coupled with halide dissociation. Using this pathway, we have developed a nickel-catalyzed stereoselective radical addition to dehydroalanine, facilitating the synthesis of noncanonical peptides. In this application, chiral ligands modulate the stereochemical outcome through the asymmetric protonation of a nickel-enolate intermediate.

The capture of the alkyl radical by nickel(II) expands the scope of cross-coupling, promotes reductive elimination through the formation of high-valent nickel(III) species, and governs chemo- and stereoselectivity. We discovered that nickel(II)-aryl efficiently traps radicals with a barrier ranging from 7 to 9 kcal/mol, followed by fast reductive elimination. In contrast, nickel(II)-alkyl captures radicals to form a nickel(III) species, which was characterized by EPR spectroscopy. However, the subsequent slow reductive elimination resulted in minimal product formation. The observed high diastereoselectivity of radical capture inspired investigations into *C-*aryl and *C-*acyl glycosylation reactions. We developed a redox auxiliary that readily couples with natural carbohydrates and produces glycosyl radicals upon photoredox activation. Nickel-catalyzed cross-coupling of the glycosyl radical with bromoarenes and carboxylic acids leads to diverse non-natural glycosides that can facilitate drug discovery.

Stoichiometric studies on well-defined d^8^-nickel complexes have showcased means to promote reductive elimination, including ligand association, oxidation, and oxidative addition.

In the final section, we address the influence of auxiliary ligands on the electronic structure and redox activity of organonickel intermediates. Synthesis of a series of low-valent nickel radical complexes and characterization of their electronic structures led us to a postulate that ligand redox activity correlates with coordination geometry. Our data reveal that a change in ligand redox activity can shift the redox potentials of reaction intermediates, potentially altering the mechanism of catalytic reactions. Moreover, coordinating additives and solvents may stabilize nickel radicals during catalysis by adjusting ligand redox activity, which is consistent with known catalytic conditions.

## Key References

- Lin, Q.; Diao, T. Mechanism of Ni-Catalyzed Reductive 1,2-Dicarbofunctionalization of Alkenes. *J. Am. Chem. Soc.***2019**, *141*, 17937–17948.^1^ A mechanistic study that establishes the sequential reduction mechanism for a nickel-catalyzed cross-electrophile coupling reaction and elucidates the mechanistic origin of cross-electrophile selectivity.
- Qi, X.; Jambu, S.; Ji, Y.; Belyk, K. M.; Panigrahi, N. R.; Arora, P. S.; Strotman, N. A.; Diao, T. Late-Stage Modification of Oligopeptides by Nickel-Catalyzed Stereoselective Radical Addition to Dehydroalanine. *Angew. Chem. Int. Ed.***2022**, *61*, e202213315.^2^ A method for synthesizing noncanonical peptides via diastereoselective radical addition to dehydroalanine using chiral nickel catalysts.
- Lin, Q.; Spielvogel, E. H.; Diao, T. Carbon-Centered Radical Capture at Nickel(II) Complexes: Spectroscopic Evidence, Rates, and Selectivity. *Chem.***2023**, *9*, 1295–1308.^3^ A study that provides spectroscopic evidence for radical capture by nickel, measures the rate of radical capture at nickel(II) complexes and reveals the difference in reactivity between C(sp^2^)–C(sp^3^) and C(sp^3^)–C(sp^3^) bond formation processes.
- Dawson, G. A.; Lin, Q.; Neary, M. C.; Diao, T. Ligand Redox Activity of Organonickel Radical Complexes Governed by the Geometry. *J. Am. Chem. Soc.***2023**, *145*, 20551–20561.^4^ A collection of well-defined nickel radical complexes that reveals an empirical correlation between coordination geometry and ligand redox activity.

## 1 Introduction

**Scheme 1:**
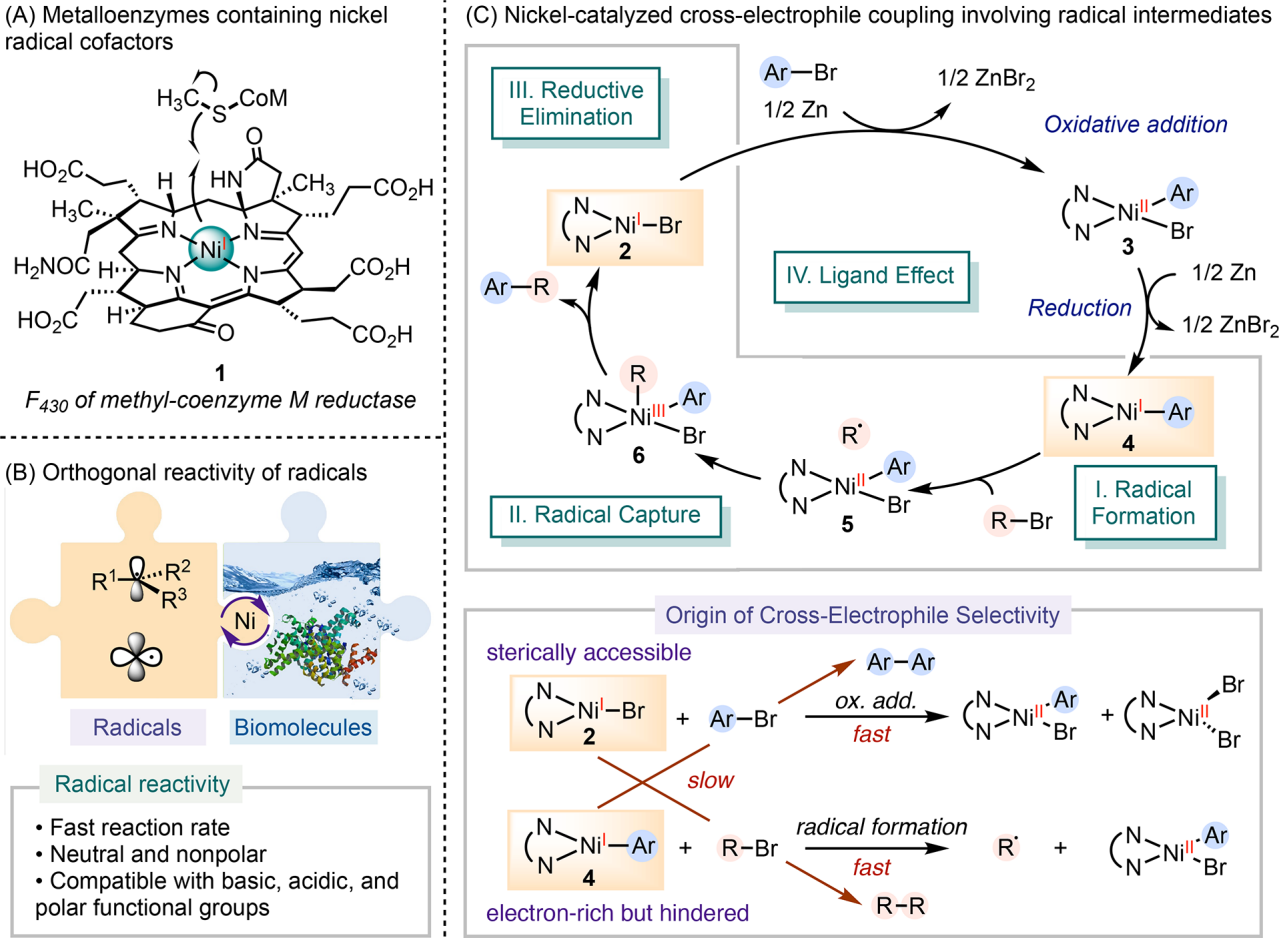
Nickel Radicals in Enzymes and Catalysis

Radical chemistry plays a vital role in biological processes, such as metabolism, replication, and biosynthesis.^5^ Many of these processes are catalyzed by cofactors featuring first-row base metals, including nickel.^6^ The relatively high pairing energy combined with weak ligand field stabilization leads to stable open-shell electronic configurations for first-row transition metal catalysts. These paramagnetic complexes are adept at initiating and propagating radical reactions through single-electron transfer (SET) and radical capture mechanisms. For instance, methyl-CoM reductase (MCR), the rate-limiting enzyme in methanogenesis and anaerobic methane oxidation, produces over a billion tons of methane annually. This massive output is achieved through SET from nickel(I) hydrocorphinate F430 cofactor **1** to methyl-CoM, resulting in the generation of a methyl radical (Scheme 1A).^7^ Other nickel-containing enzymes, including carbon monoxide dehydrogenase^8^ and nickel superoxide dismutase,^9^ respectively catalyze the reversible oxidation of CO to CO_2_ and the disproportionation of superoxide anion radical to molecular oxygen and hydrogen peroxide.

Metalloenzyme-catalyzed radical reactions highlight the orthogonal reactivity of radicals and the abundant polar functional groups found in biomolecules.^10^ While the reactivity of organic radicals are often perceived as overly reactive, their nonpolar and neutral nature make them compatible with the dominant nucleophilic and basic functional groups in biomolecules (Scheme 1B).^11^ This compatibility can confer chemo- and site-selectivity, evident in recent advancements in late-stage modification of proteins and DNA, achieved through transition metal catalysis,^12^ photoredox catalysis,^13,14^ and electrocatalysis.^15^

Over the past two decades, the rapid growth in synthetic methodology has been propelled by the application of nickel catalysts, due to their ability to mediate radical reactions.^16,17^ Modern advancements in cross-coupling reactions have broadened the scope, unlocking new routes to quickly access complex molecular architectures found in agrochemicals, pharmaceuticals, and materials. Yet, despite seminal contributions both historically and in recent times, a comprehensive mechanistic understanding of nickel-catalyzed transformations remains elusive.^18^ This article summarizes our efforts, alongside many others in this field, to unravel the fundamental reactivity of nickel-mediated radical processes, elucidate ligand effects, and develop radical reactions suitable for modifying biomolecules, including peptides and carbohydrates.

A notable synthetic application of nickel catalysis is the cross-electrophile coupling reaction.^19^ By utilizing two electrophiles, these methods circumvent the necessity for air-sensitive nucleophiles, paving the way for an extensive scope of coupling partners with good functional group compatibility. Our recent study delineated that a cross-electrophile coupling reaction proceeds through a sequential reduction mechanism (Scheme 1C).^1^ The preference for cross-electrophile coupling over homocoupling stems from the distinct activation of C(sp^2^) and C(sp^3^) electrophiles by two separate nickel(I) species, nickel-halide **2** and nickel-aryl **4**, via oxidative addition^20^ and a radical mechanism, respectively. Our program centers around dissecting each radical-involved step: (I) radical generation from the interaction of Ni(I)-aryl **4** with C(sp^3^)-halides; (II) radical capture by Ni(II) **5**; and (III) reductive elimination from nickel(III) **6**. Following each mechanistic revelation, we showcase synthetic applications that leverage the insights garnered from these fundamental steps. In the later part of the article (IV), we summarize the redox activity of *N-*ligands and their significance in nickel-catalyzed reactions proceeding through radical mechanisms.

### 1.1 Nickel(I)-Mediated Radical Formation

#### 1.1.1 Ni(I)-Mediated Radical Formation via Concerted Halogen-Atom Dissociation

**Scheme 2:**
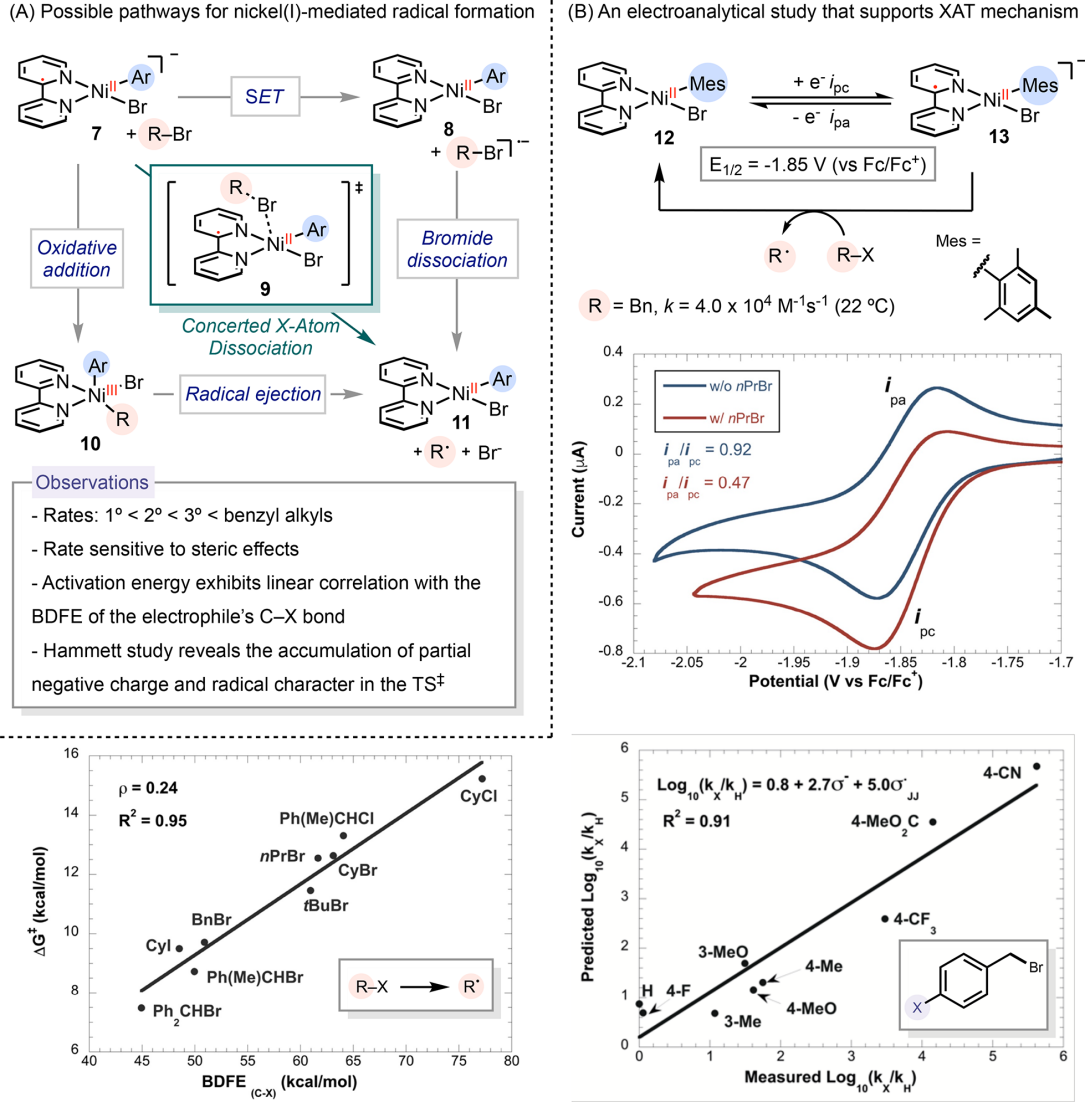
Electroanalytical Determination of a Concerted Halogen-Atom Dissociation Pathway

The activation of alkyl electrophiles by a low-valent nickel species can generate an alkyl radical. This mechanism paves the way for the cross-coupling of alkyl electrophiles and attaining stereoconvergence.^21,22^ Generally, three pathways are postulated: a stepwise SET followed by halide dissociation,^23^ an S_N_2 oxidative addition followed by radical ejection,^24^ or a concerted halogen atom transfer (XAT) (Scheme 2A).^25^ Our prior studies differentiated these pathways by investigating a series of (Xantphos)Ni(I)-aryl model complexes, supporting a concerted halogen atom abstraction pathway.^26^ However, characterizing this crucial step with catalytically relevant Ni(I) intermediates remains challenging due to their instability.

To address the challenge of studying catalytically active intermediates that are inherently unstable and thus difficult to isolate, we employed an electroanalytical method. This approach enabled the in situ generation of [(bpy)Ni(Mes)(Br)]^•–^**13** (bpy = 2,2′-bipyridine, Mes = 2,4,6-mesityl) through the electroreduction of (bpy)Ni(Mes)(Br) **12**, which allowed for the investigation of the reactivity of **13** with alkyl halides to form radicals (Scheme 2B).^27^ Cyclic voltammetry (CV) revealed a reversible **12**/**13** redox couple, evident by an *i*_pa_*/i*_pc_ ratio of 0.92 (*i*_pa_ = peak anodic current, *i*_pc_ = peak cathodic current), suggesting the stability of **13** on the CV timescale. In the presence of an electrophile, the redox event shifted from reversible to quasi-reversible or irreversible, as evidenced by a decrease of the *i*_pa_/*i*_pc_ ratio. This change was attributed to the consumption of **13** through its reaction with the alkyl halide. By varying scan rates, we determined the time course and the reaction rates across different alkyl halides. A linear free-energy regression analysis revealed a positive correlation between the bond dissociation free energy (BDFE) of the C–X bond in the electrophile and the activation barrier. Furthermore, when varying the para*-*substituents on benzyl bromides, a Hammett analysis uncovered the buildup of partial negative charge and radical character at the benzylic position in the transition state.

Benzylic and tertiary halides reacted with **13** faster than secondary and primary variants. Increasing the steric hindrance of the aryl group on nickel substantially reduced the rates of electrophile activation. Collectively, these data support a concerted inner-sphere electron transfer (ISET)/halide dissociation mechanism in the generation of radicals from alkyl halides mediated by [(bpy)Ni(Mes)(Br)]^•–^**13**. Additionally, this study rules out the outer-sphere electron transfer and S_N_2 oxidative addition pathways.

#### 1.1.2 Nickel-Catalyzed Late-Stage Modification of Oligopeptides

**Scheme 3:**
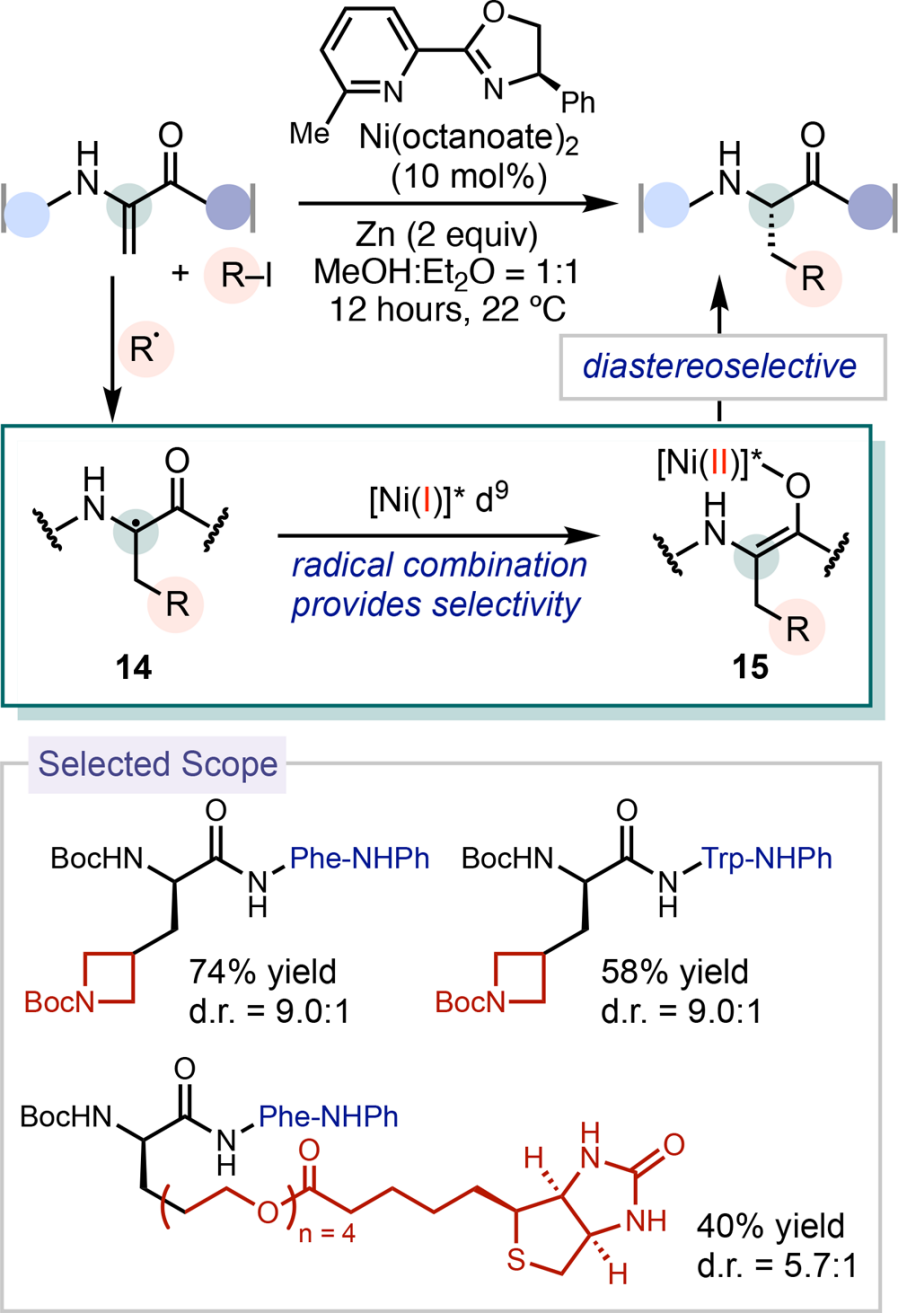
Late-Stage Functionalization of Dha via Nickel-Catalyzed Radical Addition

The ability of low-valent nickel to initiate radical formation by halogen-atom abstraction prompted us to investigate the application of this fundamental reactivity. Radical addition to dehydroalanine (Dha) provides a versatile and modular approach for synthesizing noncanonical peptide analogues, which are crucial for drug discovery.^12,28^ Previous studies on radical addition to the Dha residue of peptides and proteins have demonstrated excellent functional group compatibility.^12^ However, the lack of stereocontrol diminished the broader application of this reaction in preparing peptide analogues.^12^ To address this challenge, we postulated that chiral nickel catalysts might offer a solution for the diastereoselective late-stage modification of Dha in oligopeptides (Scheme 3).^2^ We proposed that the generation of a radical under reductive nickel-catalytic conditions could be followed by its efficient addition to Dha, resulting in intermediate **14**. A subsequent capture by a nickel(I) metalloradical species would produce enolate **15**. Given the fast rate of radical combination, this step would ensure chemo-selectivity. The enolate **15** could then undergo asymmetric protonation to establish the desired chiral center under the stereocontrol of a chiral ligand bound to nickel.

We identified a catalytic condition composed of Ni(octanoate)_2_ and the chiral ligand, (*S*)-6-Me-^Ph^pyrox (6-Me-^Ph^pyrox = 4-phenyl-2-(6-methyl-2-pyridyl)oxazoline).^2^ This system proved effective in promoting diastereoselective radical addition to Dha in dipeptides. We selected alkyl iodides as electrophiles, given their facile reactivity in forming radicals through reduction by zinc or by a low-valent nickel catalyst. This reaction framework has accommodated a variety of primary and secondary electrophiles, enabling the integration of polyethylene glycol, biotin, halo-tag, and both hydrophobic and protected hydrophilic side chains onto dipeptides. Notably, catalyst control predominantly dictates the stereochemical outcome, overcoming substrate control. Despite the versatility of this reaction, limitations remain with hydrophilic side chains and longer peptides.

### 1.2 Ni(II)-Mediated Radical Capture

#### 1.2.1 Carbon-Centered Radical Capture at Ni(II) Complexes

**Scheme 4:**
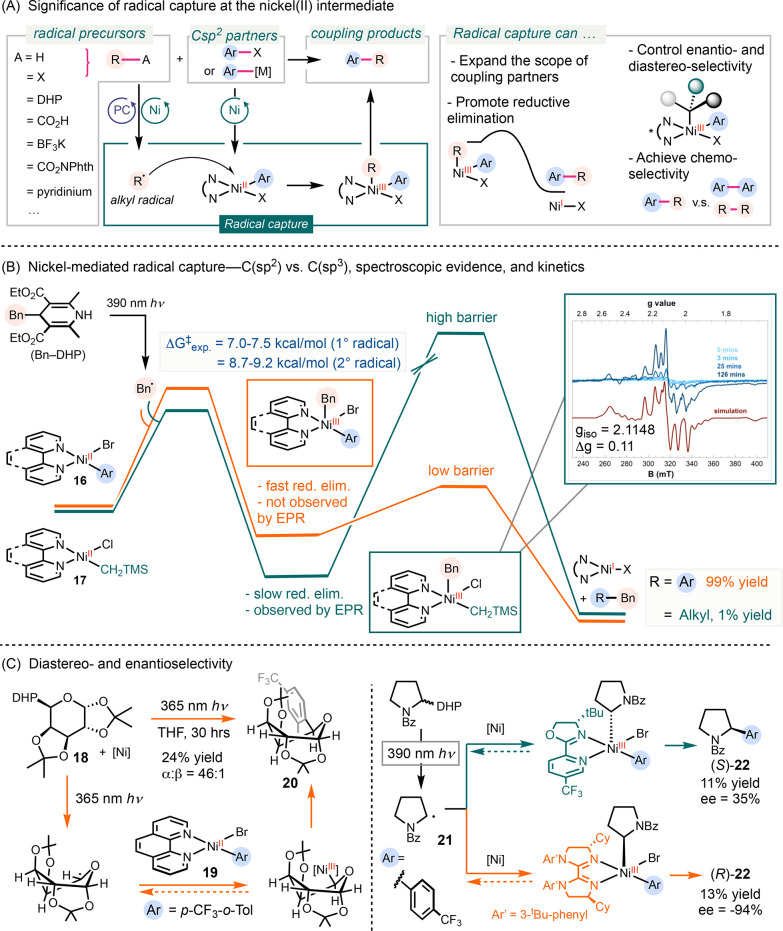
Experimental Evidence and Energy Barriers for Radical Capture at Ni(II) Complexes

The capture of carbon-centered radicals at the nickel(II) center has been widely applied in recent cross-coupling,^29^ metallaphotoredox,^30^ and electrocatalytic reactions (Scheme 4A).^31^ This step has expanded the scope of potential coupling partners to novel radical precursors, such as C–H bonds, carboxylic acids, boronates, dihydropyridine (DHP), and other redox auxiliaries.^32,33^ The formation of a high valent Ni(III) intermediate facilitates reductive elimination both kinetically and thermodynamically.^34,35^ Furthermore, the trapping of radicals by nickel complexes determines chemoselectivity in cross-electrophile coupling reactions^1^ and, with chiral ligands, controls stereoselectivity in diastereoselective and enantioselective coupling reactions.^21,22^

Previously, the capture of radicals by catalytically relevant nickel complexes has been studied computationally. These DFT calculations provided a range of kinetic barriers for this process, which vary based on the type of carbon radicals involved.^36^ The capture of primary and secondary radicals was calculated to be an inner-sphere and fast process, while tertiary radicals favor a slower outer-sphere pathway to form the C–C bond. In these computational studies, the estimated fast radical capture made it unlikely for this step to be considered rate-limiting when compared to the subsequent reductive elimination. However, there has been limited experimental characterization of radical capture at catalytically competent nickel(II) complexes that would allow for a comparison with computational results.^37^ We conducted experimental investigations into radical capture at various catalytically relevant nickel(II) complexes, determining the kinetic barriers. Our findings suggest that this step might indeed be rate-determining when compared to reductive elimination.

We investigated the stoichiometric capture of carbon radicals by a variety of nickel complexes,^3^ applying a previously reported radical precursor, benzyl-DHP (DHP = dihydropyridine), to generate benzyl radical upon photoexcitation (Scheme 4B).^38^ While bpy, phen, and pybox-ligated nickel-aryl complexes **16** (phen = 1,10-phenanthroline, pybox = pyridine-2,6-bis(oxazoline)) underwent efficient radical capture and reductive elimination, the corresponding nickel-alkyl complex **17** produced only trace amounts of product. Monitoring the addition of benzyl radical to (phen)Ni(*p*-CF_3_-*o*-Tol)(Br) (*p*-CF_3_-*o*-Tol = 2-methyl-4-(trifluoromethyl)phenyl) **16** using EPR spectroscopy revealed a signal that was consistent with benzyl radical. In contrast, performing the same experiment with (dtbpy)Ni(CH_2_TMS)(Cl), dtbpy = 4,4′-di-*tert*-butyl-2,2′-bipyridine) **17** resulted in a rhombic EPR signal with a *g*_iso_ value of 2.1148, which was assigned to a nickel(III) species. The difference in EPR signals for reactions with (phen)Ni(*p*-CF_3_-*o*-Tol)(Br) **16** and (dtbpy)Ni(CH_2_TMS)(Cl) **17** highlights the stark contrast in stoichiometric reactivity between nickel-aryl and nickel-alkyl complexes undergoing radical capture.

These results reveal different rate limiting steps for nickel-aryl and nickel-alkyl complexes. In the case of nickel-aryl complexes, radical trapping is the rate-determining step leading to the accumulation of benzyl radical, while the subsequent reductive elimination occurred rapidly. For nickel-alkyl complexes, efficient radical capture leads to the formation of an observed nickel(III) species, but the subsequent C(sp^3^)–C(sp^3^) bond-forming reductive elimination is slow (Scheme 4B).

Employing a series of radical clock experiments, we investigated the kinetics of radical capture at nickel-aryl complexes. The rates were determined to be on the scale of 10^7^ M^–1^ s^–1^ and 10^6^ M^–1^ s^–1^ for primary and secondary radicals, respectively. The corresponding activation energies were 7.0–7.5 kcal/mol for primary radicals and 8.7–9.2 kcal/mol for secondary radicals. Changing the ligands from phen to dtbpy resulted in no significant difference in the rates. These data serve as a benchmark for evaluating the kinetic competence of radical capture as an intermediate step in designing catalytic reactions.

Furthermore, we evaluated the ability of the radical capture step to impart both diastereo- and enantioselectivity (Scheme 4C). Upon irradiating **18** in the presence of 1 equiv of **19**, the glycosyl arene product **20** was obtained in 24% yield with a d.r. (d.r. = diastereomeric ratio) of 46:1. This result underscores the potential for achieving diastereoselectivity through the trapping of a glycosyl radical by nickel(II) intermediates. Subsequently, we investigated the enantioselectivity during the capture of α-amino radical **21** by nickel(II) complexes bearing chiral pyrox (pyrox = pyridine-oxazoline) and bi(imidazoline) ligands. The α-amino radical **21** proceeded to generate cross-coupling product (S)-**22** and (R)-**22**, controlled by the respective ligands. While the reversibility of radical trapping by these nickel complexes remains a question, our findings emphasize the pronounced influence of ligands in determining enantioselectivity.

#### 1.2.2 Synthesis of *C*-Glycosides via Diastereoselective Radical Capture by Nickel

**Scheme 5:**
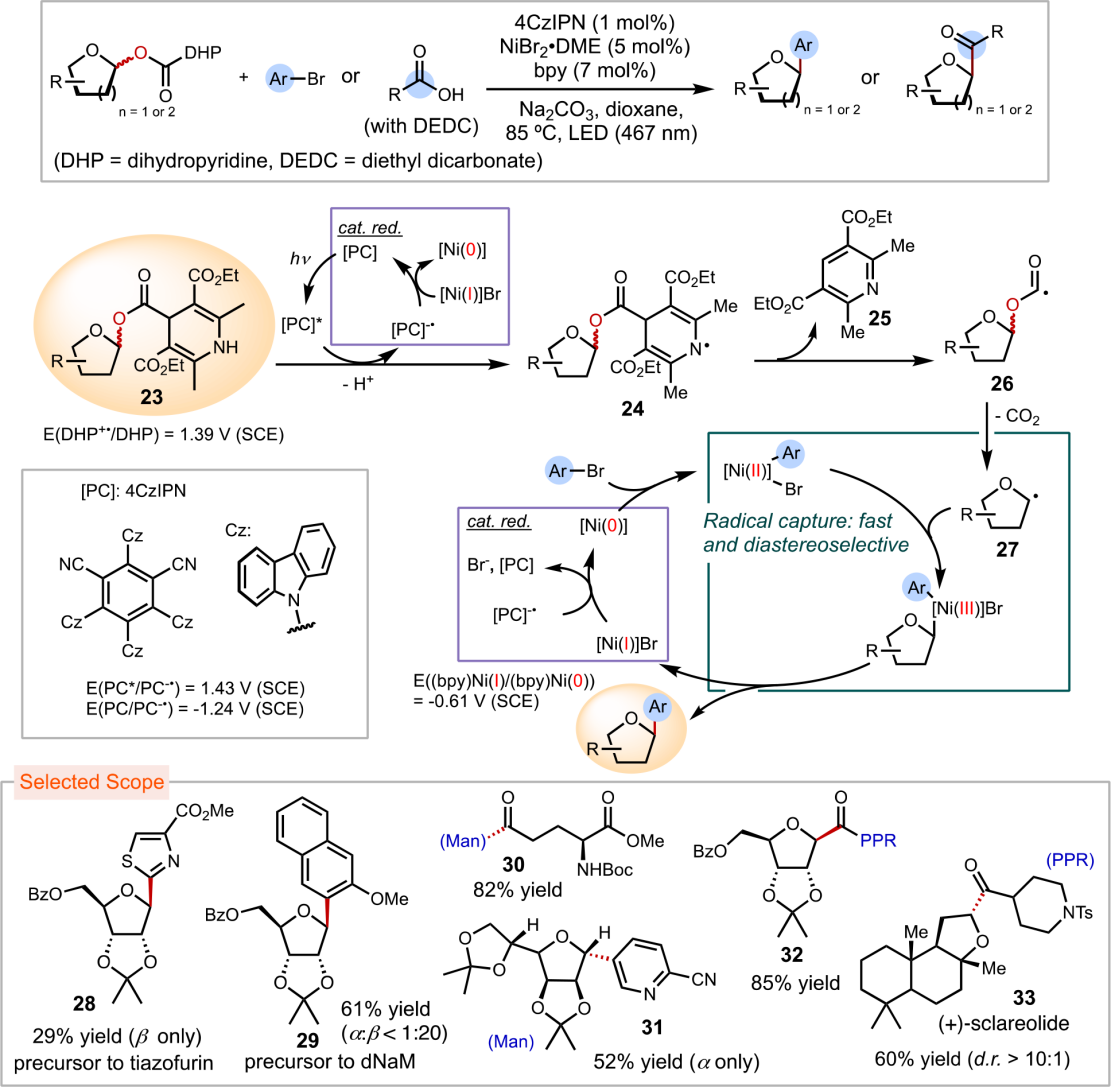
Nickel-Catalyzed *C*–Aryl and *C*–Acyl Glycosylation

Our observation of high diastereoselectivity in nickel-mediated radical capture motivated us to leverage this observation and develop a method for synthesizing *C-*glycosides from nickel-catalyzed cross-coupling of glycosyl radicals (Scheme 5).^39,40^*C*-aryl glycosyl compounds are prototypical drug candidates due to the in vivo stability of the glycosyl C–C bonds, which are resistant to hydrolysis and enzymatic degradation.^41^ Furthermore, synthetic *C*-aryl nucleoside analogues play a crucial role in examining mutagenicity origins and understanding replication and evolution mechanisms.^42^

To facilitate the formation of glycosyl radicals, we designed a redox auxiliary in the form of a DHP carboxylic acid to activate natural sugars. This auxiliary easily condenses with carbohydrate molecules, resulting in glycosyl ester **23**. This promotes glycosyl C–O bond homolysis, leading to the formation of glycosyl radicals through a sequence of photoredox oxidation (**23** → **24**), Hantzsch pyridine extrusion (**24** → **26**), and decarboxylation from the alkoxycarbonyl radical intermediate **26** (**26** → **27**). Once the glycosyl radical **27** is generated, it can promptly engage in the nickel-catalyzed cross-coupling cycle, producing aryl and acyl radical precursors as indicated by our mechanistic analyses.

We achieved efficient cross-coupling of glycosyl esters with bromoarenes and carboxylic acids applying metallaphotoredox conditions, with 4CzIPN (4CzIPN = 1,2,3,5-tetrakis(carbazol-9-yl)-4,6-dicyanobenzene) as the photocatalyst, NiBr_2_·DME (DME = 1,2-dimethoxyethane) as the catalyst, and bpy as the ligand.^39,40^ This reaction proved effective in transforming a diverse scope of furanoses and pyranoses into *C*-aryl and *C*-acyl glycosides **28**−**33**. Notably, ribose derivatives **28** and **29** synthesized via this method serve as precursors to tiazofurin, an anticancer and antiviral drug, and dNaM, a non-natural nucleoside, respectively. Additionally, a (+)-sclareolide derivative was conveniently modified with carboxylic acids to afford **33**.

Besides the products illustrated in Scheme 5, the reaction scope encompasses common furanose and pyranoses, including d-xylofuranose, d-glucofuranose, d-galactofuranose, d-arabinofuranose, d-mannopyranose, 2-deoxy-d-glucopyranose, l-rhamnopyranose, and 2-deoxy-d-ribopyranose, with various protecting groups, such as acetonide, benzyl, silyl, and benzoyl groups. For furanoses, the d.r. is dependent on C-2 substituents: incoming groups tend to approach from the face opposite to the substituent to reduce steric hindrance. In pyranosides, the d.r. is dictated by steric hindrance at C2 and the kinetic anomeric effect.^43^ However, challenges remain with 2-deoxyribose, d-glucopyranoses and d-galactopyranose, all resulting in low d.r., due to contradictory preferences by the steric and the stereoelectronic effect.

The electrophile range includes a variety of electron-rich and electron-deficient bromoarenes and heteroarenes. Regarding the carboxylic acid scope, the method proved versatile, showing compatibility with natural acids, such as fenbufen, ursodeoxycholic acid, l-glutamic acid (Glu) **30**, α-d-galactopyranuronic acid, d-biotin, and mycophenolic acid. The conjugation of Glu with carbohydrates presents an exciting avenue for glycopeptide synthesis. Given the convenient synthesis of glycosyl esters, their bench-stability, and the wide array of accessible structures, this method holds significant potential in medicinal chemistry.

### 1.3 Reductive Elimination

Various strategies have been practiced in promoting reductive elimination from a metal center.^44^ Ligand association or dissociation can lead to odd-numbered coordinate geometries, such as trigonal planar or square pyramidal. This change in geometry lowers the barrier for transferring two electrons to the vacant d-orbital. While this is a kinetic effect, reductive elimination may also be promoted by methods that exploit thermodynamic effects. Incorporating electron-deficient or bulky ligands, or oxidizing the metal center can increase the driving force by destabilizing the starting material and stabilizing the resulting low-valent metal species.^45^ Seminal reports on reductive elimination from bpy supported dialkyl nickel complexes have displayed means to trigger reductive elimination by both these kinetic and thermodynamic driving forces through oxidation, thermolysis, and ligand association.^46,47^

**Scheme 6:**
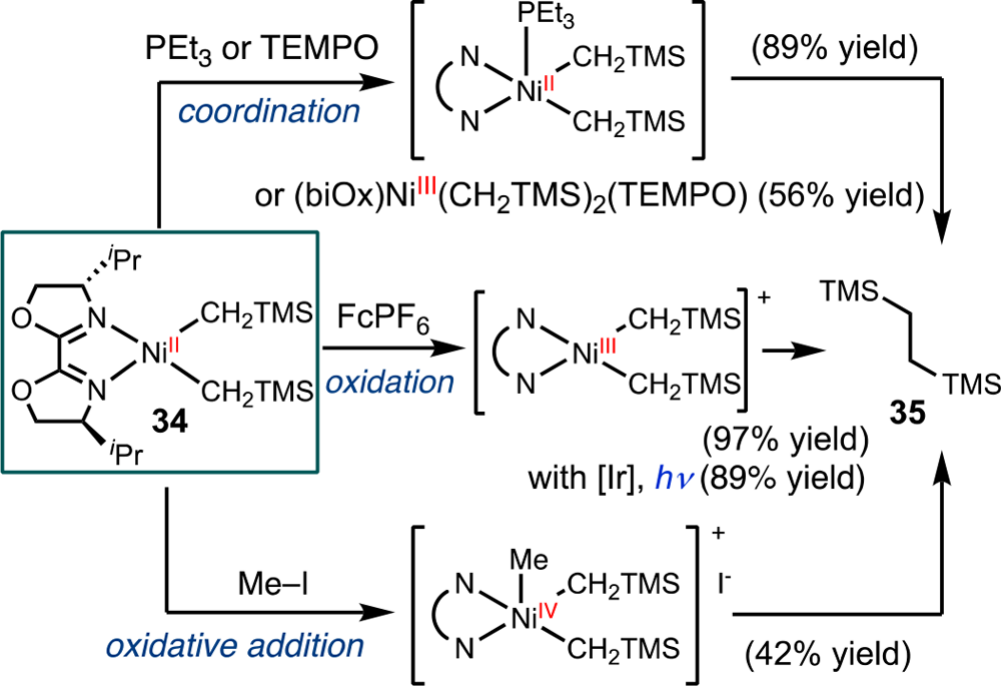
Strategies for Promoting Reductive Elimination from (^*i*Pr^biOx)Ni(CH_2_TMS)_2_

We further contributed to these aspects by investigating strategies to induce reductive elimination from a (biOx)Ni(CH_2_TMS)_2_ (biOx = bi(oxazoline)) model complex **34** (Scheme 6).^48^ The coordination with neutral donor ligands, such as triethylphosphine, and radical ligands, such as TEMPO, triggered reductive elimination to afford **35** in high yields. The oxidation of **34** using a mild oxidant FcPF_6_ (FcPF_6_ = ferrocenium hexafluorophosphate), resulted in nearly complete conversion to **35**. This oxidation can also be achieved using a photosensitizer under light irradiation. Additionally, we discerned that oxidative addition with methyl iodide generated a high-valent nickel intermediate that rapidly proceeded to reductive elimination. A concurrent study demonstrated that increasing the steric effect of the ligand can accelerate reductive elimination from a bis-alkyl nickel complex.^49^

### 1.4 Effect of *N-*Ligands

The evolution of ligands has facilitated advancements in transition metal catalysis. Phosphine and *N*-heterocyclic carbene ligands are frequently applied in cross-coupling reactions proceeding through a two-electron Ni(0)/Ni(II) catalytic cycle,^50^ whereas *N*-chelating ligands are most effective in cross-coupling reactions involving radicals.^18^ Tridentate *N*-ligands, including terpyridine (terpy) and pybox, along with diamine ligands, are effectively utilized in cross-coupling of C(sp^3^) partners.^51^ Bpy and phen ligands, meanwhile, are commonly applied in cross-electrophile coupling reactions.^19^ Chiral oxazoline ligands, such as pyrox, biOx, and bis(oxazoline) (box), impart enantioselectivity.^52^ α-Diimine ligands are proficient in promoting polymerization and hydrogenation reactions.^53^

**Scheme 7:**
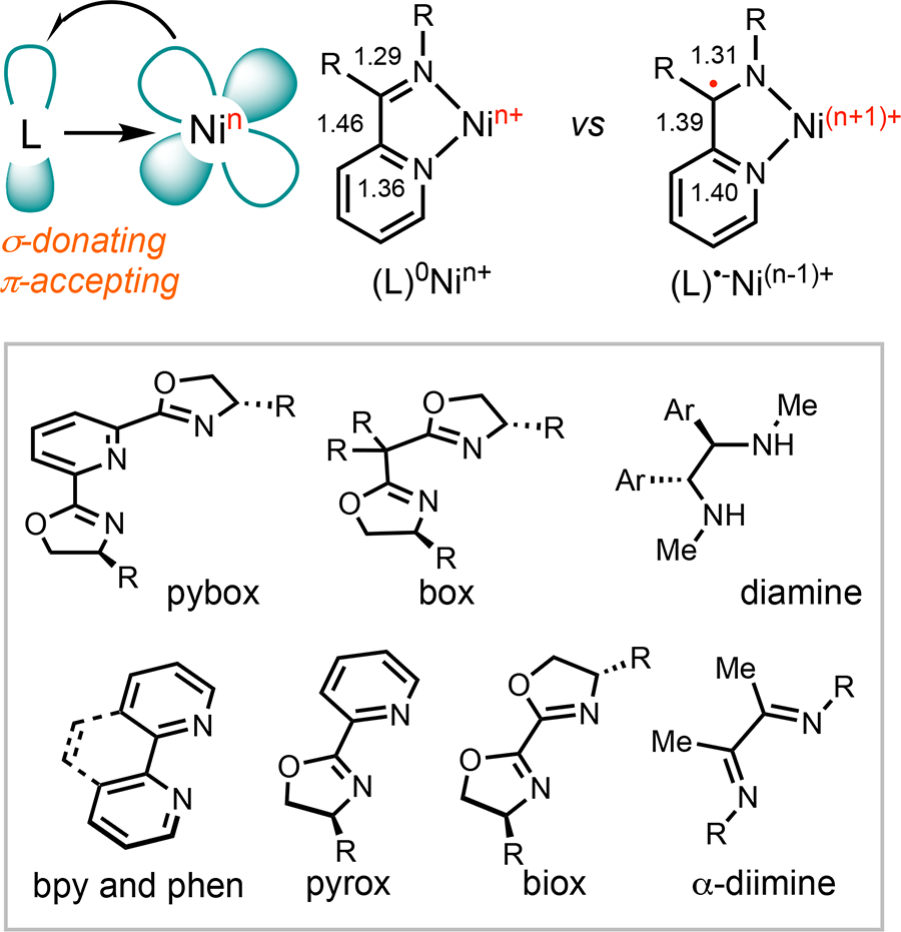
*N*-Ligands in Nickel Catalysis with Potential Redox Activity

Polypyridine ligands and related variants are strong σ-donors and π-acceptors, leading to strong field-splitting and stabilization (Scheme 7). Many of these ligands exhibit redox activity, resulting in the transfer of electrons from the metal to the π* orbital of the ligand.^54^ This effect is assessed and characterized using spectroscopic and computational methods.^55^ When a radical occupies the ligand’s π* orbital, it leads to a change in bond order. This change manifests in variations in bond lengths, which can be measured using X-ray diffraction (XRD) or predicted through DFT calculations. EPR spectroscopy can reveal the nature of the radical, distinguishing between ligand-centered and nickel-centered radicals. Since the valence orbitals determine the redox potentials of a complex, the redox activity of the ligand can have profound effects on reaction pathways. Consequently, discerning the electronic structures of reaction intermediates becomes crucial to understanding the ligand effect, guiding ligand design for optimizing reactions.

#### 1.4.1 Redox Activity of Pyrox in Organonickel Radical Complexes

Pyrox, with its extensive applications in asymmetric catalysis,^56^ often functions similarly to bpy or phen in catalyzing coupling reactions. Prior to our study, the redox activity of pyrox and its comparison to other *N*,*N*-ligands remained ambiguous.

**Scheme 8:**
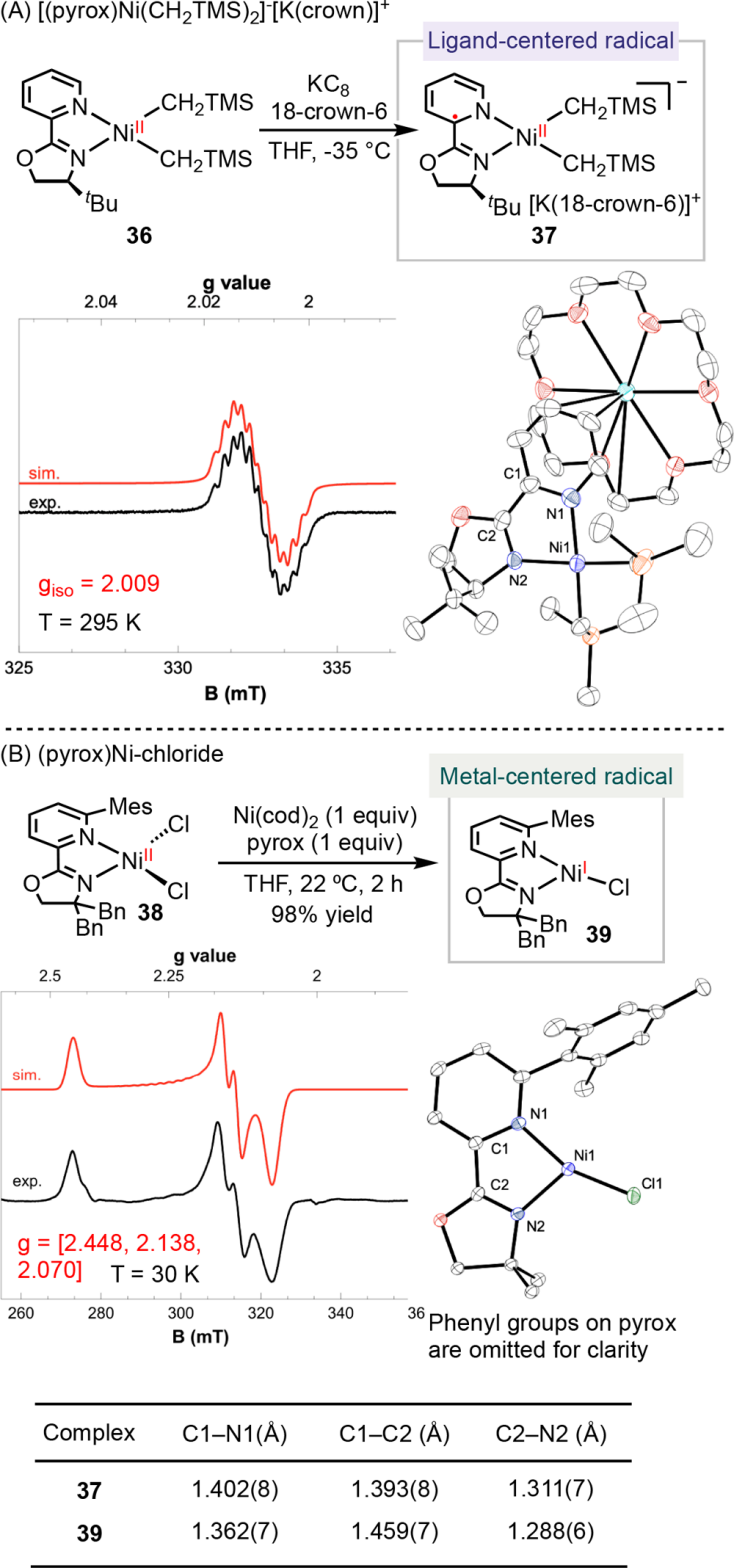
Redox Activity of (Pyrox)Ni Complexes

We investigated the electronic structures of a series of pyrox-ligated nickel radical complexes, using two synthetic strategies. In the first approach, the reduction of (^*t*Bu^pyrox)Ni(CH_2_TMS)_2_**36** (^*t*Bu^pyrox = (S)-4-(*tert*-butyl)-2-(2-pyridyl)oxazoline) by potassium graphite (KC_8_) in the presence of 18-crown-6 led to the formation of [K(18-crown-6)]^+^[(^*t*Bu^pyrox)Ni(CH_2_TMS)_2_]^•–^**37** (Scheme 8A).^57^ The second approach involved the comproportionation of (pyrox)NiCl_2_**38** and Ni(cod)_2_, giving (pyrox)NiCl **39** in 98% yield (Scheme 8B).^4^ X-ray crystallography established unsymmetrical Ni–C bond lengths in **37** and a T-shape geometry in **39**, highlighting a pronounced trans-influence of pyridine compared to oxazoline. The elongation of C_ox_–N_ox_ and C_py_–N_py_ bond lengths, combined with the contraction of the C_ox_–C_py_ bond, suggests a radical in the π* orbital of **37**. This assignment is corroborated with EPR spectra, which reveals a ligand-centered radical in **37** and a nickel-centered radical in **39**. Collectively, these data imply that that the redox activity of pyrox varies depending on the coordination environment of each complex.

#### 1.4.2 Bi-Oxazoline Organonickel Radical Complexes

**Scheme 9:**
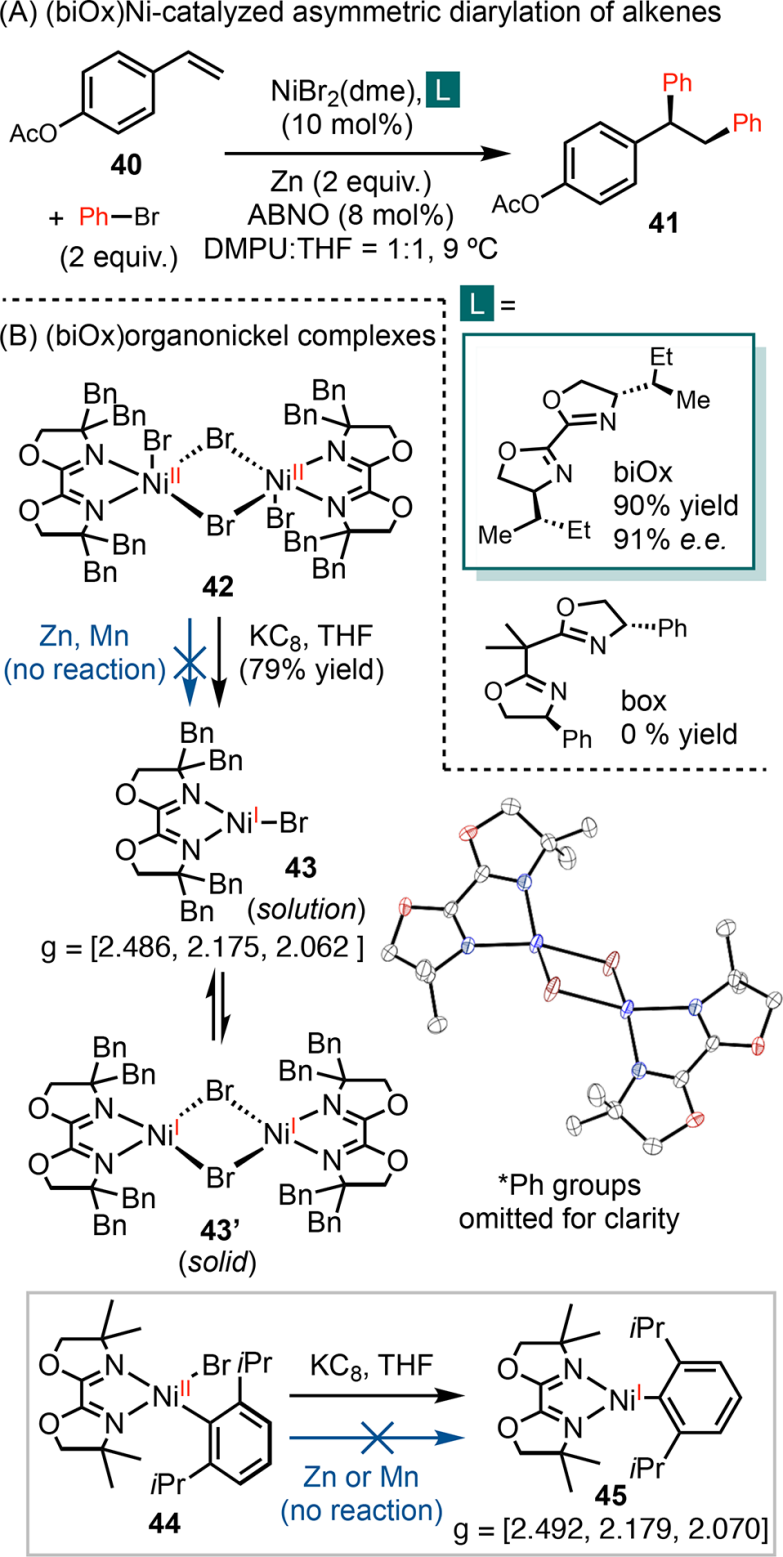
Application and Redox Activity of (biOx)Ni Complexes

BiOx ligands exhibit intriguing reactivity in asymmetric catalysis, particularly with nickel catalysts.^51^ For instance, we achieved high yields and enantioselectivity with biOx in the development of an asymmetric diarylation of vinylarenes (Scheme 9A).^58^ In contrast, using the structurally similar box ligand resulted in no product formation.

To elucidate the ligand effect of biOx, we synthesized organonickel complexes and characterized their electronic structures (Scheme 9B).^59^ The reduction of (^dBn^biOx)NiBr_2_**42** yielded (^dBn^biOx)NiBr **43**, which exists in equilibrium with the dimer [(^dBn^biOx)Ni(μ-Br)]_2_, **43′**. The EPR spectrum of **43** reveals an *S* = 1/2 nickel complex as the predominant species in solution, whereas the X-ray crystal structure displays a dimer **43′**, with a tetrahedral geometry at the nickel center. Reduction of (^dMe^biOx)Ni(Dipp)Br (Dipp = 2,6-diisopropylphenyl) **44** by KC_8_ afforded **45**, characterized as a nickel-centered radical by its EPR signal. Both the EPR data and bond lengths derived from XRD suggest an absence of redox activity in biOx.

**Scheme 10:**
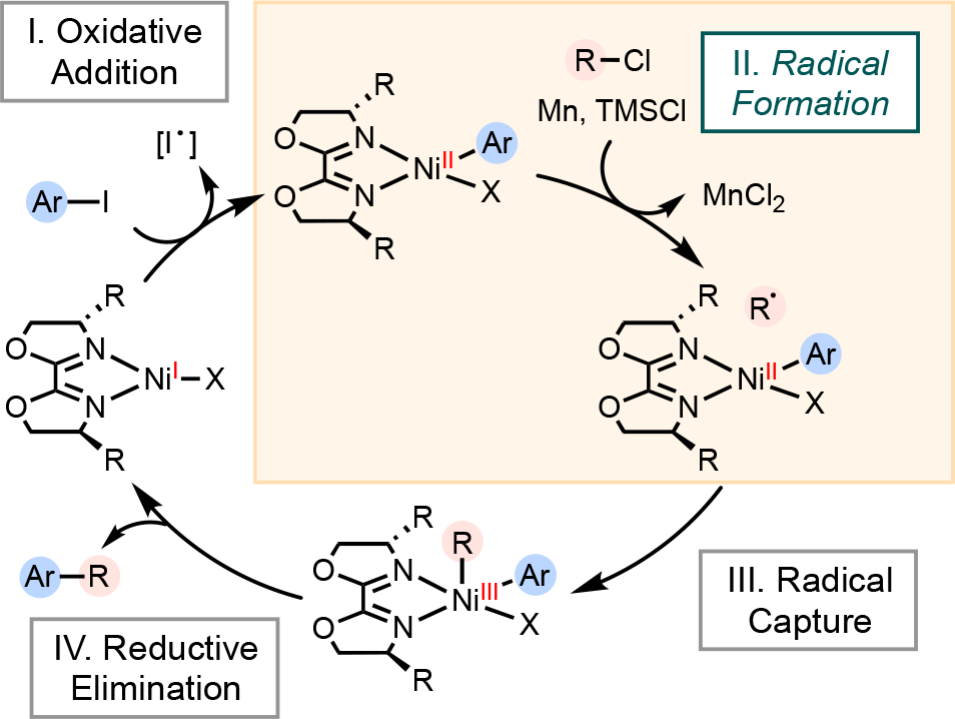
Revised Mechanism of Cross-Electrophile Coupling Involving (biOx)Ni Catalysts

The lack of redox activity in these biOx complexes could be attributed to the π-donating effect of the *O*-atom of oxazoline. The elevated energy level of the ligand π*-orbital prevented electron transfer from nickel to the ligand. As a result, (biOx)nickel complexes exhibit significantly lower reduction potentials compared to their phen, bpy, and pyrox analogues (Table 1).^60^ When we tried to synthesize **43** and **45** through reduction, neither zinc nor manganese powder—common reductants in cross-electrophile coupling—initiated any reaction (Scheme 9B). This observation led us to re-evaluate the mechanism of cross-electrophile coupling reactions involving (biOx)Ni catalysts and proposed that a catalyst reduction step was not operational in a reaction catalyzed by (biOx)nickel catalysts (Scheme 10). Instead, our revised proposal suggests a direct interaction between substrates and reductants, facilitated by a Lewis acid, to generate radicals.

**Table 1:** Comparison of the Redox Activity and Redox Potentials of (*N*,*N-*Bidentate)Ni(Aryl) Complexes

Ni complexes	redox activity	*E*_1/2_ [Ni(II)/(I)] (V vs Fc^+^/Fc)
(phen)Ni(Mes)Br^61^	yes	–1.50
(bpy)Ni(Mes)Br^62^	yes	–1.79
(^*t*Bu^pyrox)Ni(Dipp)Br^56^	yes	–1.35
(^dMe^biox)Ni(Dipp)Br 45^58^	no	–2.12

#### 1.4.3 Geometry-Dependent Redox Activity in Nickel Radical Complexes

Our study on (pyrox)Ni complexes indicates that the redox activity of a ligand can vary among different complexes (Scheme 8). This observation remains consistent across a range of reported organonickel radical complexes ligated with bidentate *N*-ligands.^4^ By analyzing data from the literature combined with our results, we established a general framework for understanding the ligand redox activity of nickel radical complexes.^4^ These complexes feature ligands that are commonly employed in cross-coupling reactions: such as bpy, phen, and pyrox. This empirical rule entails a correlation between the ligand redox activity and the coordination geometry of the complexes.

**Scheme 11:**
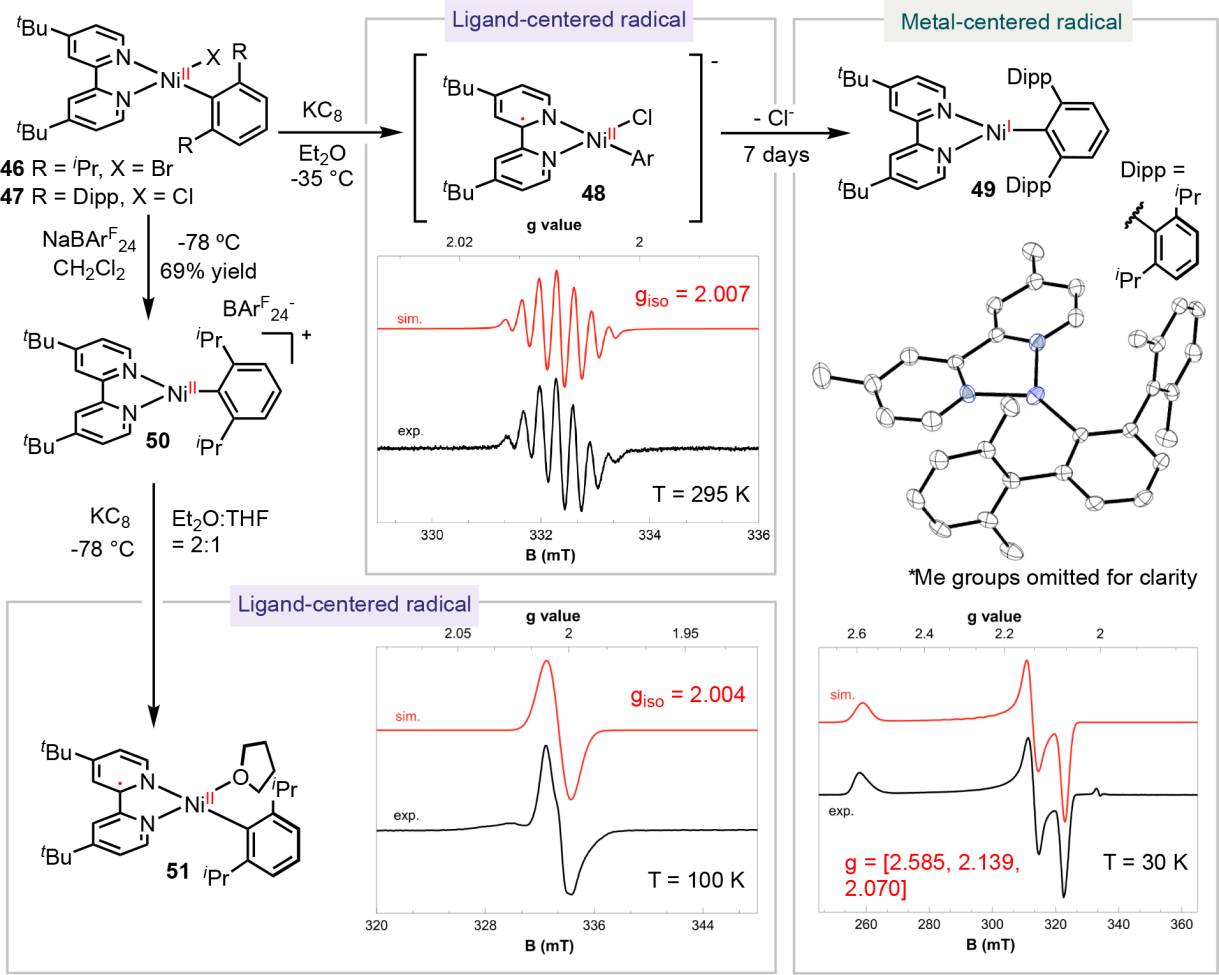
Effect of Coordination Geometry on the Redox Activity of (Bpy)Ni-Aryl Complexes

We synthesized and characterized a series of (bpy)Ni radical complexes. The reduction of (dtbpy)Ni(Dipp*)Cl **47** (Dipp* = 2,6-di-Dipp-phenyl) led to the formation of complex **48**, which was characterized as a ligand-centered radical by EPR (Scheme 11).^4^ Over time, in solution, chloride dissociation led to the formation of the three-coordinate complex **49**, which was identified as a metal-centered radical based on its EPR spectrum. Furthermore, when we reduced [(dtbpy)Ni(Dipp)]^+^[BAr^F^_24_]^−^ (BAr^F^_24_ = tetrakis(3,5-bis(trifluoromethyl)phenyl)borate) **50** in THF, we observed an isotropic, organic radical. Intriguingly, performing this reduction in a less coordinating solvent, such as Et_2_O, yielded a three-coordinated nickel radical **56**, showing a rhombic EPR signal with g values of [2.263, 2.086, 2.050].

**Scheme 12:**
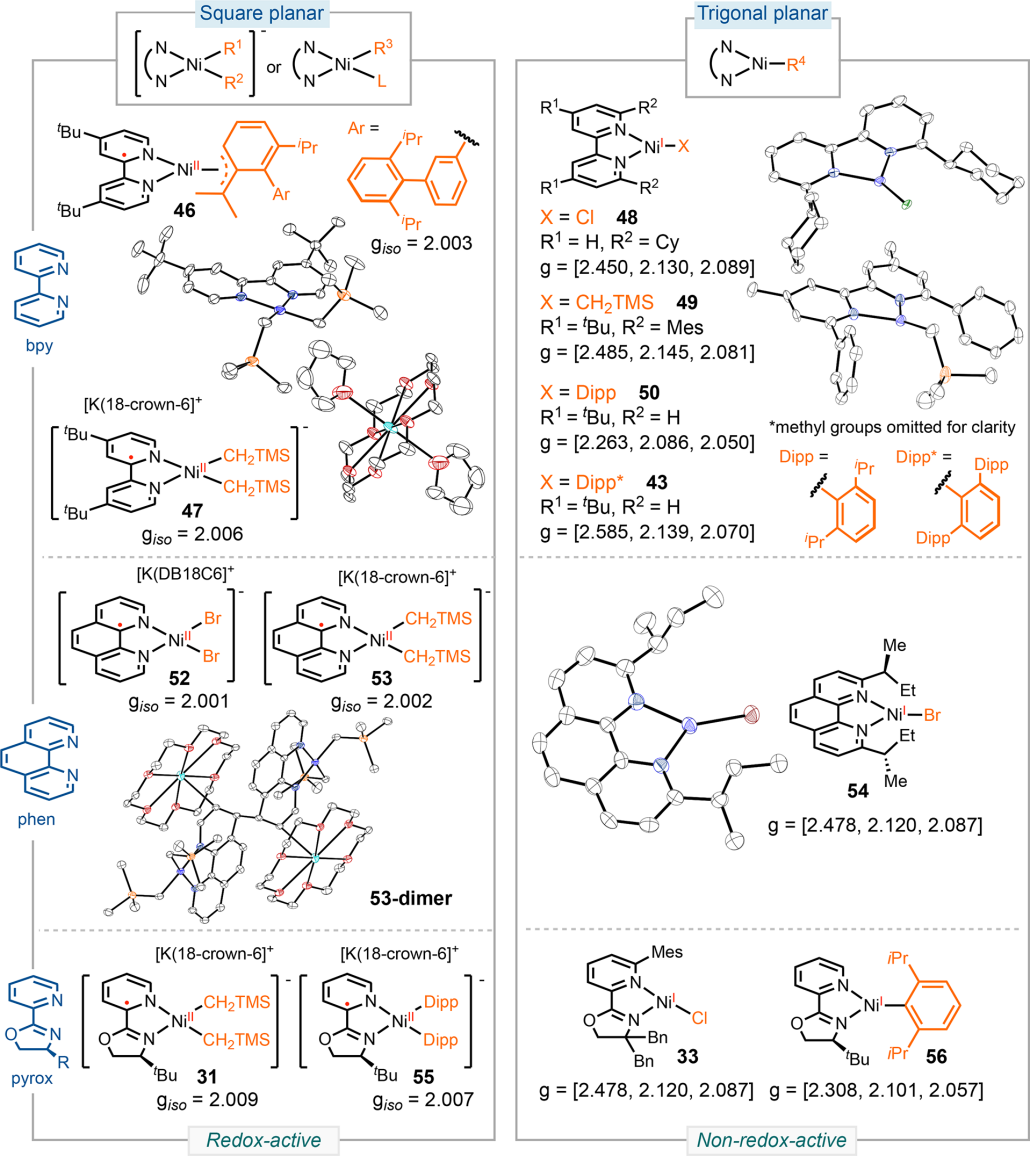
Redox Activity of bpy, phen, and pyrox Ligands in Nickel Radical Complexes and the Correlation with Coordination Geometry

By increasing the steric hindrance of the bpy ligands and the X-ligand, we synthesized a series of (bpy)Ni radical complexes (Scheme 12).^4^ The (π-allyl)Ni **52** and the square-planar [(dtbpy)Ni(CH_2_TMS)_2_]^−^[K(18-crown-6)]^+^**53** are characterized as ligand-centered radicals. In contrast, the trigonal planar (^Cy^bpy)NiCl **54** and (^Mes^dtbpy)Ni(CH_2_TMS) **55** display metal-centered radicals, evidenced by the lack of significant changes in bond lengths and the rhombic EPR signals corresponding to a nickel-centered radical. Nickel radical complexes with phen ligands follow a similar trend. The four-coordinate [(phen)NiBr_2_]^−^[K(DB18C6)]^+^**57** (DB18C6 = dibenzo-18-crown-6) and [(phen)Ni(CH_2_TMS)_2_]^−^[K(18-crown-6)]^+^**58** are determined to be ligand-centered radicals based on EPR and XRD analyses, whereas the three-coordinate (^sBu^phen)NiBr **59** is a nickel-centered radical.^1^

Overall, both the literature data and our results point to a correlation between ligand redox activity and coordination geometry. Four-coordinate, square-planar nickel radical complexes are characterized as low-spin nickel(II) complexes coordinated with ligand radical anions.^63^ In contrast, tetrahedral nickel radical complexes, such as **43′**, as well as three-coordinate, trigonal planar nickel-halide, alkyl, and aryl complexes typically exhibit nickel(I) centers, supported by non-redox-active ligands. This trend persists irrespective of the nature of the X-ligands or the charge of the molecule across a range of nickel complexes with bpy, phen, pyrox, α-diimine, and biox ligands, characterized by our group and documented in the literature. Notably, (terpy)Ni complexes are exceptions to this trend.^64^

**Scheme 13:**
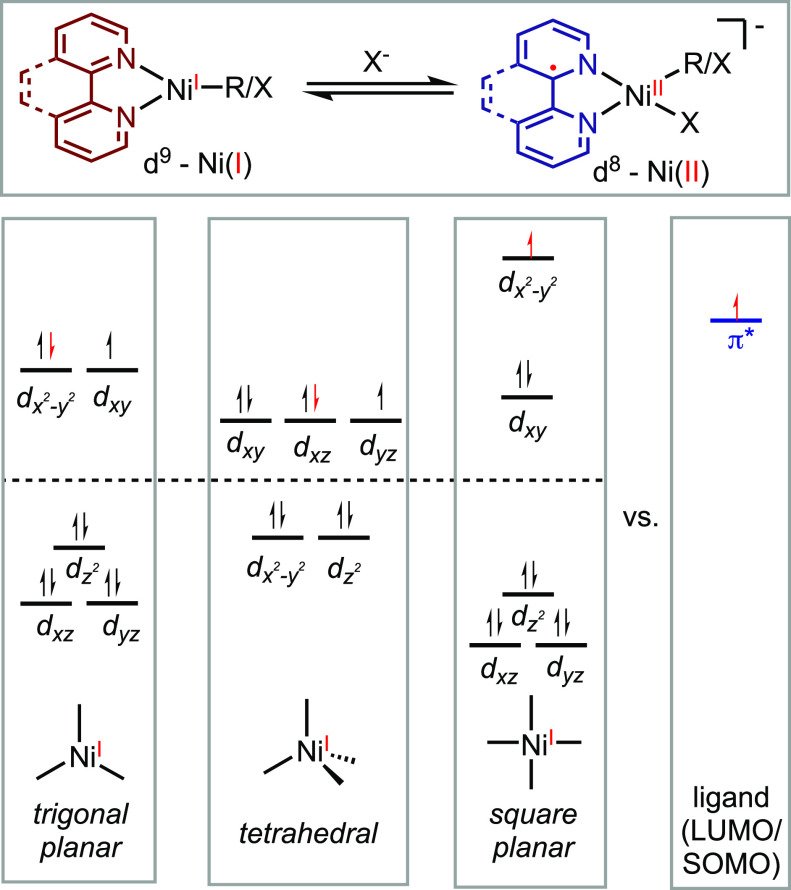
Geometry-Governed Ligand Redox Activity

This relationship between ligand redox activity and coordination geometry can be understood through molecular orbital analysis (Scheme 13). The redox activity of a ligand depends on the relative energy levels of the vacant d-orbital and the π* orbital of the ligand. Molecules with trigonal planar and tetrahedral geometries have low-energy antibonding d-orbitals, often lying beneath the ligand’s π* orbital. As a result, it is energetically favorable for the unpaired electron to remain in the d-orbital, resulting in a d^9^ electronic configuration. Conversely, for square planar complexes, the high energy level of the d_*x*2-*y*2_ orbital implies a preference for the unpaired electron to transfer to the ligand π* orbital, leading to a d^8^ configuration.

Our findings provide insights to help optimize catalytic conditions. Nickel-catalyzed cross-coupling reactions often apply additives such as MgCl_2_ and KI. Besides their role in modulating the speciation of active nickel catalysts,^65^ the coordinating anions may be pivotal for the stabilization of nickel(I) intermediates and the modulation of redox potentials through the alteration of ligand redox activity. Furthermore, our results imply that coordinating solvents may promote reactions through a similar stabilization effect.

## 2 Conclusions

Nickel catalysts excel at initiating carbon radical formation and engaging radical intermediates in cross-coupling reactions. We have investigated the mechanisms, kinetics, and ligand effects on fundamental steps that involve radical intermediates. The nonpolar and neutral reactivity of these radical intermediates, orthogonal to polar functional groups in biomolecules, has enabled the diastereoselective modification of dehydroalanine for preparing noncanonical peptides. Furthermore, our study on radical capture by nickel has led to the discovery of a series of *C*-glycosylation reactions, useful for preparing *C*-aryl and *C*-acyl glycosides valuable in drug discovery.

Our continued focus revolves around elucidating the mechanisms of nickel-mediated and catalyzed reactions that involve radical intermediates. We are particularly interested in understanding these pathways at the interface of photoredox catalysis and electrocatalysis. Central to catalyst development is ligand design. Compared to the vast library of phosphine ligands available for palladium, the variety of *N*-π-acid ligands for nickel are disproportionally insufficient. We are actively exploring and developing new ligands to broaden the scope of nickel catalysis. While the application of the orthogonal reactivity of radicals with polar functional groups in biomolecules remains in its early stages, our methodology projects aim to bridge this knowledge gap and implement methods for accessing noncanonical peptide and nucleoside analogues.
